# The Changing Educators’ Work Environment in Contemporary Society

**DOI:** 10.3389/fpsyg.2018.02186

**Published:** 2018-11-13

**Authors:** Monica Pedrazza, Sabrina Berlanda, Federica De Cordova, Marta Fraizzoli

**Affiliations:** Department of Human Sciences, University of Verona, Verona, Italy

**Keywords:** educator’s work environment, transformations in educational work environment, social educator, attachment style, self-efficacy, job satisfaction

## Abstract

In this paper, we are going to address job satisfaction and perceived self-efficacy withinthe context of residential child-care. A joint report from the European Foundation for the Improvement on Living and Working Conditions and the European Agency for Safety and Health at Work revealed that managers in the field of health and education were the most concerned about the psychosocial risk of their employees, although concern is not automatically translated into tools to face the risk and to manage it. So, measuring and improving employees’ job satisfaction and self-efficacy can be an important means for organizations to prevent the outcomes of psychosocial risk, and supporting high quality performance of workers. But profound changes are affecting the nature of work at large, and specifically social educator’s in the field of residential care with minors. Globalization, radical technological and communication developments, as well as the pressure to frame care as a commodity, are quickly changing procedures and praxis at work, and even the meaning of job itself. All these changes are highly demanding for this category of professionals, as much as the fact that the organizational setting is vanishing as a resource to sustain their professional attitudes and behaviors. Under these circumstances, job satisfaction and self-efficacy can be hard to experience, and isolating their precursors is essential to develop healthy and effective work environments. This paper means to highlight the process of supporting self-efficacy and job satisfaction in the educational work in residential youth care that is still underrepresented in research. It presents data emerging from two studies, study 1 involving 268 educators and study 2 involving 472 educators belonging to different Italian residential child-care services. Study 1 consists of a quantitative study including the following measures: attachment style, job satisfaction, work-related self-efficacy, and length of service. Study 2 consist of a qualitative exploration deepening the sources of educators’ work-satisfaction. Quantitative data support the identification of attachment style and length of service as antecedents of work-related self-efficacy and job satisfaction. Qualitative data show the importance of relational issues in shaping the educators’ satisfaction at work.

## Introduction

Job satisfaction and perceived self-efficacy are two main components supporting the more general sense of fulfillment and well-being at work, negatively correlated with psychosocial risk. Fostering positive conditions at work is considered by the European Union crucial in containing the psychosocial risk of workers, making it a pivotal prerequisite to accomplish within the 2020 Agenda goals on employment. Improving work conditions, and job satisfaction among them, is a guarantee of keeping workers involved in the labor market (Eurofound and Eu-Osha, 2014).

In 2014, a joint report from the European Foundation for the Improvement on Living and Working Conditions and the European Agency for Safety and Health at Work revealed that managers in the field of health, social work and education were the most concerned about the psychosocial risk of their employees, although concern is not automatically translated into tools to face the risk and manage it (Eurofound and Eu-Osha, 2014). So, measuring and improving employees’ job satisfaction and self-efficacy can be an important means for organizations to prevent the outcomes of psychosocial risk, and supporting high quality performance of workers (Singhai et al., 2016).

In this paper, we are going to address job satisfaction and perceived self-efficacy within the context of care work in child and youth care. Indeed, a better understanding of the impact of the work of care on employees’ fulfillment puts professionals working in the abovementioned areas in a better position compared to managers and professionals belonging to different sectors. Nonetheless, profound changes are affecting the nature of job at large, and specifically social educator’s in the field of residential care with minors. Globalization, radical technological and communication developments, as well as the pressure to frame care as a commodity, are quickly changing procedures and praxis at work, and even the meaning of job itself. All these changes are highly demanding for this category of professionals, as much as the fact that the organizational setting is vanishing as a resource to sustain their professional attitudes and behaviors. Under these circumstances, job satisfaction and perceived self-efficacy can be hard to experience, and isolating their precursors is essential to develop healthy and effective work environments.

The sample choice has been deliberately strong in methodologically terms, since it refers to a group of workers in a high stress (Pedrazza and Berlanda, 2016), high demand field undergoing an unusual situation adding to this already difficult field (Pedrazza and Berlanda, 2016). We deemed that studying these psychological phenomenon (attachment, job satisfaction, self-efficacy) with a group under greater than normal stress could help us to determine the interaction among factors in a more clear way.

### Job’s Structural Changes and the Phenomenon of Unaccompanied and Separated Children

Research focusing on variables that facilitate or, conversely, hinder positive experience at work is massive (Lambert et al., 2002; Lee and Cummings, 2008; Judge et al., 2010) and nowadays we are aware that an array of factors affects work satisfaction. Although intrinsic job qualities (security, career, contract) have a direct impact in determining it, for most people the meaning of work goes far beyond, and there is evidence that the relational work environment as well as individual characteristics play a significant role too (Manisera et al., 2005; Eurofound, 2012).

However, contemporary society is in the middle of an epoch-making turn bringing about shifts in the models of production and consumption, and in the social structure and organization (Bauman, 2002; Appadurai, 2013). In such scenario, work is in the front line: job insecurity, poor contract terms, optimization, delayering, ever-changing working conditions and related skill-building, high level of turnover, short term goals, technological innovation, represent some of the issues for the workers to be mastered. In some cases, it means potential opportunities for self-fulfillment and job satisfaction or, more often, the origin of emotional and psychological distress (Sennett, 1998; Eurofound, 2015).

The literature suggests that massive organizational changes challenge workers to maintain a good sense of identity going through such transformations (Woods, 2008); in doing so, trust seems to play a role in mediating the perception of the self in relation to the organization (Sloyan and Ludema, 2010). Wherever the case, the way change is implemented and especially, the organization’s capabilities in reducing stress levels and assisting workers in coping with increased demands, are key factors in predicting successful adjustment to organizational change (Tvedt et al., 2009).

One very specific feature shaping the direction of change characterizing contemporary Western society is migrations, that continue to represent a significant challenge across Europe. Data updated to 2015 report that in Italy 48% of minor population in residential care is of non-Italian (Autorità Garante per l’Infanzia e l’Adolescenza, 2017). Another relevant phenomenon within this group is the number of unaccompanied and separated children (UASC). Twenty thousand of these children reached Europe in 2017 and four out of five got to Italy, where 86% of children of foreign origin arriving every year are UASC. Italy offered international protection to 16,309 unaccompanied children in 2017, in youth residential care units (UNHCR, 2017). Although the trend of arrivals is decreasing as a consequence of the drop in the number of people crossing the Mediterranean, such proportions in youth population in residential care units are stable. For unaccompanied refugee children, Italy is both the main target for the disembarkation (others are Spain, Greece, and Bulgaria) and for settlement (others being Germany, France, and Greece) (UNHCR, 2017).

We lingered over the phenomenon of unaccompanied children to highlight how much it affects the work of social educators in youth residential care. For a long time, it has been representing a very challenging field of employment because of the difficult nature of the work and the high demands and expectations placed on workers (Annie E. Casey Foundation, 2003). These children hold multiple needs, requiring specific strategies in taking charge and proposing interventions. And, generally speaking, multiple, and complex reasons lead children (both of Italian and foreign origins) to have access to these structures (Autorità Garante per l’Infanzia e l’Adolescenza, 2017). Education workers in these contexts are therefore presented with a set of unique tasks that require the constant management of a wide degree of diversity and the capacity to operate primarily in unpredictable and emergent situations rather than planned settings. Consequently, there have been calls from a number of directions for specialized training and the development of innovative competences that can be deployed in the face of recent social transformations and the need for different ways of working, partly to increase the quality of the educational intervention itself, but also to reduce the level of burnout among these workers (Servizio centrale del Sistema di protezione per richiedenti asilo e rifugiati, 2015).

### The Work of Social Educators

If these claims regard work conditions at large, we want to focus now on the peculiarity of the social educator’s work in the field of child and youth care. The professional knowledge establishing the social educational practice has been defined by various sciences such as developmental and social psychology, ethics, anthropology, sociology. The methods are multidimensional and include: care, education, intervention, treatment, development of non-exclusive life space treatment.

Drawing on previous attempts by different national associations of social educators to arrive at an agreed definition of the common traits that define the work of the social educator, we can identify one of the most salient aspects in the role of “the relationship” as professional tool, whereby making the educator’s own body and mind provide the core of his or her professional resources. This specificity demands commitment: without this premise, no relationship can be achieved (European Bureau of the International Association of Social Educators [AIEJI], 2006).

Social educators work is therefore based on “being in action,” in the relationship, using themselves to support the child development. Such actions are rooted in conscious deliberations converted into a planned and target-oriented process. Social educational action is devoted to the adult life, (the mentally disordered, alcohol or drug abusers, homeless people, etc.) although it originates with children and young people (European Bureau of the International Association of Social Educators [AIEJI], 2006).

The centrality of relationship exposes social educators to the children’s needs as they are responsible for responding to them in order to support the development of their personal skills. In doing so, social educators are the frontline staff, the first to cope with children’s crises, anger, aggression, and interpersonal conflict resolution (Zerach, 2013). But they are also the frontline staff in face of the social changes. As we have seen with the example of migrations, educational work is at the foremost for detecting movements and transformation in the society. Because of this very peculiar position, educational professionals working with minors in residential care have to be continuously innovative in their practice.

Another core skill in child and youth residential care regards the capacity to deal with intense and possibly disturbing emotional issues (Shinn et al., 1993; Shapiro et al., 1996; Williamson, 1996; Smith, 2005; Smith and Shields, 2013). This competence demands the professional to find a balance between – on the one hand – their closeness to, and empathy toward the child’s experience, and – on the other – their awareness that they, the educator, are “someone else,” a separate person with their own needs and feelings (Golia and Pedrazza, 2014; Golia et al., 2017; Berlanda et al., 2018). The centrality of the relationship as well as the engagement with the job can be the basis of the experience of job satisfaction and perceived self-efficacy. However, such emotional labor is highly energy consuming and sometimes the organizational, interpersonal and intrinsic characteristics of the work environment can fail to sustain the employee. The result can be disruptive for the individual, ending up with job stress, burnout, and intent to leave (Cetrano et al., 2017).

Furthermore, current changes in both the working culture of the organization and the specific role of the worker, of the sort we have already mentioned, can lead the educator to find this relational aspect of the job intimidating. Yet, most of the workers find intrinsic value in their work, experience job satisfaction and continue in their perception of self-efficacy (Smith and Shields, 2013). Faced with this ambivalent data, our hypothesis is that in the absence of the structures once provided by the organization, and lacking any form of mediator, educators are left feeling exposed and isolated in having to form and manage the educational relationship, and that it is therefore worthwhile investigating which other resources are available.

Traditionally, the concept of burnout (Maslach, 1976, 1982) has been applied to care work to account for the role (and the failure) of the organizational variables in mediating the effect of job stressors on the individual, as well as in fostering job satisfaction and perceived self-efficacy. According to this theoretical model, organizational variables can be extremely powerful in positively mediating the personal resources involved in experiences of work and, conversely, their ineffectiveness is a major cause for burnout. It is a fact that the organizational features that were considered risk factors in Maslach’s theoretical model of burnout (Leiter and Maslach, 1988), have come to characterize a large number of today’s work environments (Gallie et al., 2012; Gosetti and La Rosa, 2014). This can certainly be considered the case with the risk of overexposure to disturbing emotional issues that characterize the helping professions.

A different frame is offered by the compassion satisfaction/fatigue model (Figley, 1995, 2002). In this case, the same stimulus (exposure to the client’s heavy psychological sufferance) can provoke compassion fatigue, and the consequent negative effects on the professional (anxiety, depressive symptoms, avoidance, relational problems, cognitive shifts); or, on the contrary, the professional’s experience can be described in terms of compassion satisfaction, that means perceived as extremely challenging and rewarding by the professional. This alternation between compassion fatigue and compassion satisfaction suggests that the key variable in determining the shift from fatigue to reactivity and self-efficacy is the worker’s own personality.

Through the following research data, we aim to shed light on the process of supporting self-efficacy and job satisfaction in the educational work in residential child-care, that, despite its pivotal role in child and family services, is still underrepresented in research (Colton and Roberts, 2007).

### Job Satisfaction and Self-Efficacy at Work

Satisfied and happy workers are more productive (O’Keeffe et al., 2015; Richards et al., 2017; Semachew et al., 2017). In other words, employees who are satisfied with their jobs have better performance (Singhai et al., 2016). Given that job satisfaction is a crucial concept, it is connected with a large number of important organizational phenomena. The concept of job satisfaction is complex (Coomber and Barriball, 2007) and has a variety of definitions and related issues (Misener et al., 1996).

Job satisfaction is related to a positive attitude toward the job, and there is no universal measure for this attribute. Job satisfaction can be defined as a discrepancy between what people want in a job and what they have in a job (Locke, 1969) or as a feeling of fulfillment or pleasure associated with one’s work (Del Valle et al., 2007; Singhai et al., 2016). Job satisfaction is the extent to which employees like their jobs (Zangaro and Soeken, 2007), and whether they find them enjoyable and interesting (Kacel et al., 2005). Job satisfaction has been associated with professional, personal, interpersonal, and organizational variables (Adams and Bond, 2000; Ruggiero, 2005; Cicolini et al., 2014). Job satisfaction is considered as a multi-dimensional concept (Caricati et al., 2014; Gkolia et al., 2014; Singhai et al., 2016; Mangaleswarasharma, 2017), with each dimension having some importance (McGlynn et al., 2012). Researchers have studied different components of job satisfaction, for example Weiss and Brief (2002) have highlighted the importance of affect at work.

Job satisfaction is a central component of a person’s general well-being. Attachment theory also suggests that the attachment style significantly contributes to general subjective well-being and life satisfaction (Reizer, 2015).

Little attention has been paid to the social educators’ experience of job satisfaction. There is evidence of residential child-care workers’ job satisfaction positively impacting the experiences and emotions of the children and adolescents for whom they are responsible (Fein, 2014). Therefore, it is crucial to address the question of which factors contribute to educators’ job satisfaction.

According to Bandura (1977) self-efficacy is defined as a set of beliefs about the one’s ability to perform well at work, in specific settings and situations. Self-efficacy is not a general trait manifesting uniformly across the various domains of activity. Performance and outcomes are influenced by perceived self-efficacy in multiple ways (Bandura, 2012). Actually, efficacy perceptions guide human behavior through cognitive, motivational, affective and decisional processes. In the field of social education self-efficacy is the professional’s confidence in the ability to contribute to the wellbeing of children and fulfilling their needs (Chen and Scannapieco, 2010). A higher level of self-efficacy is related to lower emotional exhaustion, and to low occurrence of the intent to leave the work among professionals (Chen and Scannapieco, 2010).

Due to the importance of job satisfaction and self-efficacy, it is interesting to explore which factors influence them.

### Attachment Style and Length of Service

The first and foremost relational nature of the educators’ work enables us to ground the perspective from which we look at educators into “a relational based theory” (Reizer, 2015) such as the attachment theory (Ainsworth, 1978). Secure subjects score lower on neuroticism and higher on extroversion than both insecure groups. In addition, they score higher than avoidant subjects on agreeableness. Furthermore, in planning behavior within relationships, secure adults tend to integrate emotional and cognitive stances; avoidant adults tend to over rely on cognitive considerations, usually ignoring or denying emotional responses. Anxious adults tend to focus mainly on emotions issues and coping (Feeney and Noller, 1996). The personal attachment system entails internal working models of the self, of others and, last but not least, of how relationships typically work and develop based on the individual’s own primary relationships in early life. Over time, it therefore impacts and shapes our perception of every issue concerning the way we act, re-act, and interact in each interpersonal domain. Personal style attachment is relatively stable over time because of its effectiveness in shaping our expectations. Attachment style exerts its impact on interpersonal processes according to both threats’ severity and closeness of the relational bond (Ronen and Zuroff, 2017). Professionals’ secure attachment has been shown to have a positive impact on patient’s health outcomes in the long term (Mimura and Norman, 2018). Jallalmanesh et al. (2015) research results show a positive and significant relationship between attachment insecurity, burnout and occupational stress. Providing adequate caregiving and effective social support are adult behaviors, strongly depending on attachment security, that is, on the evaluation of oneself as effective and able to recognize others’ needs and on the evaluation of others as deserving help and support (Mikulincer and Shaver, 2007). Secure attached professionals are flexible and responsive in meeting others’ needs; they tend to develop empathic compassion rather than personal distress in facing suffering and vulnerable others. Attachment security has been shown to be a protective factor against emotional exhaustion and burnout (Golia et al., 2017).

Attachment security, which supports effective caregiving practices, is strengthened by both intra – and interpersonal regulation (Rholes et al., 2001; Henninghayusen and Lyons-Ruth, 2005; Vogel and Wei, 2005). In addition, adequate caregiving requires the ability to synchronize and coordinate one’s own caregiving and care seeking behaviors in order to cope with, and solve any problem at hand (Collins et al., 2006). Although differing from the typical caregiving behavior, instrumental helping, as a form of mere task – oriented interpersonal behavior (Bowler and Brass, 2006; Pedrazza et al., 2015b), also depends on the personal attachment style (Geller and Bamberger, 2009). Securely attached professionals are perceived by their co-workers as offering the highest frequency of instrumental help. Compared to insecure individuals, secure ones report a larger number of collaborative, and pro-social behaviors (Ronen and Zuroff, 2017). Moreover, according to the dependency paradox (Feeney, 2007) when leaders, supervisors, or co-workers respond appropriately during times of stress, professionals are more likely to explore, and to engage in autonomous behavior. According to Harms (2011)’ review there is a strong relationship between attachment style and a certain number of outcomes such as job satisfaction, work-family spillover, citizenship behaviors and job performance (Yip et al., 2018). Burnout and compassion fatigue are known to be high risks associated to any type of helping profession (Figley, 2002; Stamm, 2010; Pedrazza et al., 2015a). Despite differences, both concepts relate to various negative psychological outcomes associated with the provision of help and care to at-risk or vulnerable populations. Furthermore, literature shows that whereas attachment anxiety is strongly related to workplace incivility, emotional exhaustion and cynicism, attachment avoidance is negatively related to civility, psychological safety, and trust (Leiter et al., 2015).

One further important characteristic more consistently associated with job satisfaction and self-efficacy is the length of service. As workers get older they acquire greater security, they interiorize pivotal elements of the organizational knowledge acquiring tools and techniques and feeling thereby less vulnerable to job stress (Del Valle et al., 2007). Younger workers suffering from burnout may have not developed the coping skills to deal with the high levels of stress associated with child protection work (Leiter, 1990; Boyas et al., 2012). Younger workers recognize in older ones the expertise to deal with critical situations (Cloutier et al., 2012).

## Materials and Methods

Two self-report on-line questionnaires were administered to two convenience samples of educators, working in residential child-care in Northeast Italy. Because of the relevance of Italy in the current context of the migrations’ flows, it is worthwhile to examine the features and characteristics of the educators’ ever-changing working experience at the present moment. In Italy, an educator is a specific professional who helps others to acquire knowledge, competences and/or values. Educators in residential child-care are “front-line” workers, that interact with children who are placed out of their homes, and away from their families of origin. Residential child-care workers cooperate closely with social workers and teachers, child welfare officers and administrators, nurses and psychiatrists, therapists, and special educators. Educators’ work is vital because they take on the role of “surrogate parents” (Whittaker and Maluccio, 2002; Del Valle et al., 2007), and they are more likely to influence children’s behavioral development than other professionals. Educators’ role is difficult and involves multiple stressors. They have to cope with children’s crises, states of anger, frustration, aggression, and interpersonal conflict resolution. They are expected to be flexible by providing each youth with a unique relationship that provides children with nurturance and acceptance, but also need to provide them with very clear limits and boundaries (Pazaratz, 2000; Hanson, 2015).

Email addresses to hand out the questionnaires were provided by communities’ management. Ethical approval was obtained by the Human Sciences Department’s Ethics Committee (University of Verona). Questionnaires included a section that explained the nature and the purpose of the study and a consent form. Written informed consent was obtained from each participant, who voluntarily participated in the study. Participants were informed about their right to withdraw or refuse to give information at any time without incurring in any penalty. We protected the privacy and anonymity of individuals’ answers involved in our research.

The research was articulated into two studies. The studies were carried out in two different contexts and periods. The first consists of a quantitative inquiry, that was carried out in January 2017. We explored the impact of attachment insecurity and length of service on job satisfaction, we also explored the mediating effect of perceived work self-efficacy. Our aim was to identify the antecedents of job satisfaction, that latter is a sufficiently studied and analyzed concept, there are in fact a great number of measures in order to assess this variable, we therefore did not need its in-depth exploration. As we identified the antecedents we realized that the experience of satisfaction at work was in some measure predictable. We decided therefore to further explore not so much the concept of satisfaction but its sources, in order to provide educators with useful information to be implemented in their intervention on the work context and in their continuous training programs. The second, the qualitative study was carried out in April 2017.

### Study 1: A Model of Work-Related Self-Efficacy

Aim of Study 1 is to test the mediating effect of work-related self-efficacy in the relationship between length of service, attachment style, and job satisfaction.

As above mentioned, we start with the assumption that, as matters currently stand, there is a lack of organizational support available for educators, thus we assume the pivotal role of both variables of individual difference and personal characteristics in predicting job satisfaction.

H1 = There is a positive and significant correlation between length of service and job satisfaction;

H2 = There is a negative and significant correlation between attachment avoidance and job satisfaction;

H3 = Personal self-efficacy mediates the relationship between the independent variables such as attachment style and length of service, and job satisfaction (dependent variable).

#### Participants and Procedure

The questionnaire included job satisfaction, work-related self-efficacy, attachment style, and some questions on demographic and occupational characteristics (gender, age, and length of service).

The sample was composed of 268 residential child care workers. Participants were in a large majority female (186, 69.4%), 77 were males (28.7%), and 5 participants have not indicated the gender (1.9%). The mean age was 35.68 years (*SD* = 10.02; *range* = 20–63; 1 missing data, 0.4%), and the mean length of service was 9.42 years (*SD* = 7.94; *range* = 1–39; 3 missing data, 1.1%).

#### Measures

##### Job Satisfaction

Job satisfaction was measured with one item (“I am satisfied with my job”). Responses were given on a 7-point Likert scale ranging from 1 (complete disagreement) to 7 (complete agreement).

##### Work-Related Self-Efficacy (Barbaranelli and Capanna, 2001)

Self-efficacy was assessed by the Perceived Personal Efficacy for members of volunteering associations. This instrument includes 18 items, assessing the extent to which members of associations feel capable of facing different situations, critical events and challenges occurring during their everyday activity (e.g., “I am able to handle the stress of my job,” “I am able to cooperate with my colleagues”). Responses were given on a 7-point Likert scale ranging from 1 (complete disagreement) to 7 (complete agreement). The reliability of the scale was α = 0.909.

##### Experiences in Close Relationship Scale (ECR)-Short Form (Wei et al., 2007)

Six items measured Anxious attachment (e.g., “My desire to be very close sometimes scares people away,” “I need a lot of reassurance that I am loved by my partner”), and 6 items measured Avoidant attachment (e.g., “I want to get close to my partner, but I keep pulling back,” “I try to avoid getting too close to my partner”). Responses were given on a 7-point Likert scale ranging from 1 (complete disagreement) to 7 (complete agreement). Cronbach’s alphas were 864 (Anxious attachment) and 887 (Avoidant attachment).

#### Data Analysis

In order to test the mediation effect of self-efficacy, the bootstrapping procedure (Hayes, 2013) was used. Following this procedure, three regression analyses were conducted. First, the mediator (self-efficacy) was regressed on the independent variables (length of service, anxious attachment, and avoidant attachment). Second, the dependent variable (job satisfaction) was regressed on the independent variables. Finally, the dependent variable was regressed simultaneously on both the mediator and the independent variables. All regressions were carried out on 5,000 resamples. The indirect effect was the product between the regression coefficient obtained in the first regression and the regression coefficient linking the dependent variable with the mediator (third regression). The indirect effect is significant, if zero is not included in the confidence interval (Hayes, 2009). The direct effect is estimated by the third regression equation. The total effect (i.e., the sum of the direct and indirect effects) of independent variables on dependent variables is estimated by the second regression equation.

### Study 2: Sources of Educators’ Job Satisfaction

Aim of the Study 2 is exploring residential child-care workers’ subjective sources of satisfaction at work.

#### Participants and Procedure

The sample was composed of 472 educators. Participants were in a large majority female (331, 76.1%), 136 were males (28.8%), and 5 participants have not indicated the gender (1.1%). The mean age was 36.73 years (*SD* = 10.16; *range* = 20–65; 5 missing data, 1.1%), and the mean length of service was 10.01 years (*SD* = 8.35; *range* = 1–40; 8 missing data, 1.7%).

The questionnaire included one open-ended question about sources of satisfaction at work (“What is your first source of satisfaction at work?”). The maximum length of the participants’ answer was 140 characters (spaces included). The questionnaire included also some questions on demographic and occupational characteristics (gender, age, and length of service).

#### Data Analysis

Qualitative analysis was performed with NVivo 11. Two researchers have independently coded and analyzed 472 sources of satisfaction at work using thematic analysis (Braun and Clarke, 2006; Clarke and Braun, 2013; Vaismoradi et al., 2013; Maguire and Delahunt, 2017). Unit of meaning was the whole answer of the respondents. Thematic analysis is a flexible and useful research tool to identify and analyze patterns in qualitative data (Clarke and Braun, 2013), which allows to capture something important about data, and “represents some level of patterned response or meaning within the data set” (Braun and Clarke, 2006: 82).

We used a semantic approach. We identified the themes within the explicit meanings of the data, and our analysis was not looking for anything beyond what a participant had written. We adopted an essentialist/realist approach, assuming that language reflects the experience of participants. We identified themes in an inductive or bottom up way (e.g., see Pedrazza and Berlanda, 2014). An inductive approach is a process of coding the data without trying to fit it into a pre-existing coding frame, and therefore this thematic analysis is essentially data driven (Braun and Clarke, 2006). We adopted a recursive process (Maguire and Delahunt, 2017). First, researchers read data in an active way searching for meanings, and then they produced initial codes. Afterward, independent judges sorted and collated units of meaning into categories generated *ad hoc* for each theme (key words or abbreviations). At this point, researchers reviewed the categories considering internal homogeneity and external heterogeneity (Patton, 2015). Researchers compared their results only when they had finished their work, in order to exclude the possibility of their influencing each other. We used Cohen’s kappa to measure intercoder reliability. We controlled intercoder reliability for 100 analyzing units (21.19%). Cohen’s kappa was calculated, using SPSS program, by comparing judgements of researcher 1 and researcher 2. In our study, moderate agreement (the lower level) is shown by the code “Achieve the children’s well-being” (κ = 0.56), and substantial to almost perfect agreement (the higher level) is shown by “Interaction with colleagues and supervisors (κ = 0.83).

## Results

### Study 1: Descriptive Statistics

The means, standard deviations, and intercorrelations between constructs are reported in Table 1. Regarding the relations between variables, results indicated that self-efficacy at work was positively correlated with job satisfaction, and negatively with avoidant attachment. Whereas length of service was positively correlated with self-efficacy at work and job satisfaction.

**Table 1:** Descriptive statistics and intercorrelations (N = 268).

		Mean	SD	1	2	3	4	5
1	Length of service	9.42	7.94	1				
2	Anxious attachment	3.09	1.07	0.083[–0.029, 0.197]	1			
3	Avoidant attachment	2.74	1.11	0.029[–0.115, 0.172]	0.235^∗∗∗^[0.102, 0.355]	1		
4	Job satisfaction	5.35	1.26	0.125^∗^[–0.007, 0.249]	0.002[–0.127, 0.124]	–0.114[–0.260, 0.033]	1	
5	Self-Efficacy	4.99	0.97	0.234^∗∗∗^[0.102, 0.351]	–0.037[–0.160, 0.080]	–0.184^∗∗^[–0.319, –0.061]	0.599^∗∗∗^[0.502, 0.684]	1

*^∗^*p* < 0.05, ^∗∗^*p* < 0.01, ^∗∗∗^*p* < 0.001.*

### Study 1: Regression Analysis

Table 2 detail the estimates and the 95% bias corrected confidence intervals. Results showed that self-efficacy was negatively related to avoidant attachment, and positively related to length of service. Regarding job satisfaction results showed that its relation with length of service was positive, whereas its relation with avoidant attachment was negative. The total effect of anxious attachment was not significant. With regard to self-efficacy, results indicated that it was positively related with job satisfaction. As for the indirect effects, results showed that self-efficacy mediated the effects of avoidant attachment; indeed, zero is not included in the confidence interval. Avoidant educators have a low level of job satisfaction, and this would be explained by their perceived work-related self-efficacy.

**Table 2:** Mediation effects of job satisfaction on self-efficacy (*N* = 268).

		Self-efficacy	Job satisfaction		Indirect effect	Bias correct 95% confidence interval	
		**β (*SE*)**	**β (*SE*)**	**β (*SE*)**	**β (*SE*)**	**Lower**	**Upper**

1	Length of service	0.030^∗∗∗^	0.020^∗^	–0.003	0.023	0.013	0.046
		(0.007)	(0.010)	(0.008)			
2	Anxious attachment	–0.012	0.024	0.033	–0.009	–0.120	0.102
		(0.056)	(0.074)	(0.060)			
3	Avoidant attachment	–0.165^∗∗^	–0.137^∗^	–0.010	–0.128	–0.294	–0.050
		(0.053)	(0.071)	(0.058)		
4	Self-Efficacy			0.773^∗∗∗^			
				(0.067)			
	*R^2^*	0.092	0.030	0.360			
	*F*	8.773^∗∗∗^	2.672^∗^	36.611^∗∗∗^			
	*df*	3,264	3,264	4,264			

*β, unstandardized coefficient; 5,000 resamples. ^∗^*p* < 0.05, ^∗∗^*p* < 0.01, ^∗∗∗^*p* < 0.001.*

### Study 2: Thematic Analysis

**FIGURE 1:**
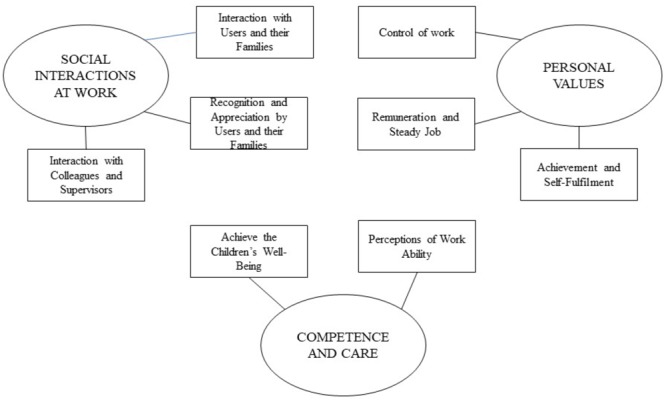
Sources of job satisfaction.

The thematic analysis identifies the pivotal sources of job satisfaction, which are consolidated under three overarching themes: Interactions (46.82%), Competence and Care (29.24%), and Personal Values (23.94%). Themes and sub-themes are presented in the thematic map of our data (Figure 1).

The theme *Social Interactions at Work* is composed by three sub-themes: Interactions with users and their families (59.73%; e.g., “I love working with children”), Interactions with Colleagues and Supervisors (28.05%; e.g., “I enjoy the relationships with my colleagues”), and Recognition and Appreciation by users and their families (12.22%; e.g., “children parents are often thankful and appreciated my work”). Relational factors that educators have identified as sources of job satisfaction are, for example, support from management and leadership, colleagues’ supportive behavior, children’s collaborative behavior, collaboration with parents and families, and perceived respect from children and their families.

The theme *Competence and Care* is composed by two sub-themes: Perceptions of Work Ability (58.70%; e.g., “The frequent social interaction at work it is a clear indication of my social ability”), and Achieve the Children’s Well-being (41.30%; e.g., “My first aim is to guarantee the children’s well-being and when I achieve this goal I feel fulfilled”). In these sub-themes educators have identified as sources of job satisfaction the perceived ability to deliver effective care, the feeling of “making a difference” in children’s lives, and the wellbeing of the children.

The theme *Personal Values* is composed by three sub-themes: Achievement and Self-Fulfillment (67.26%; e.g., “Working in education is fulfilling”), Remuneration and Steady Job (17.70%; e.g., “The steady work gives me a lot of security”), and Control of Work (15.04%; e.g., “I can organize autonomously my work-time schedule”). In these sub-themes educators have identified as sources of job satisfaction: self-fulfillment and self-actualization, creativity and opportunities to personally contribute to the decisions making process, salary and benefits.

## Discussion

In study 1 we tested the effects of self-efficacy, length of service, and attachment style on job satisfaction. Our results suggest that job satisfaction is predicted by both length of service and attachment avoidance: greater length of service is associated to perceived higher job satisfaction; higher scores in attachment avoidance correspond to lower levels of job satisfaction.

We then tested the mediating role of self-efficacy: results show that there is no mediating effect in the relationship between length of service and job satisfaction; conversely, self-efficacy mediates the relationship between attachment avoidance and job satisfaction.

According to literature (Mikulincer and Shaver, 2007) avoidant subjects do not seek relational proximity because they are afraid of closeness and intimacy. In addition, they tend to adopt distancing-based relational strategies. According to Mikulincer and Shaver (2007), and to Gillath et al. (2005) there is a negative association between avoidant attachment, caregiving and pro-social orientation. It appears therefore rather unlikely that avoidant people gain a sense of achievement in taking care of others. Nevertheless, results of the present study suggest that under the same attachment related conditions (avoidance), avoidant people who score much higher in work-related self-efficacy are more satisfied with their job. We hypothesize that the sense of efficacy regarding their work and performance may confirm and further support the positive image of themselves (Internal Working Model), which in turn allows them to feel more satisfied (higher levels of job satisfaction) even in handling with vulnerable and needy others. The present study indicates a rather affordable resource for supporting avoidant educators to enhance their work-related self-efficacy and job satisfaction. It is now quite clear that sources and core perceived components of job satisfaction have to be explored in order to support the development of both professionals’ and educative third-sector organizations.

There is no effect of attachment anxiety on educators’ self-efficacy and job satisfaction. Being the relational domain the topos where the educators’ professionalism may manifest itself, the absence of any effect may be due to the fact that anxious subjects typically feel threatened by any relational concern or problem. Nevertheless, their fear of rejection also forces them to be hyper-vigilant and thereby to disengage from work-based relationships in order to quickly solve problems and to cope with low self-esteem (Geller and Bamberger, 2009). In addition, the recurrent preoccupation for others may hinder anxious people to acknowledge any positive result and outcome.

The second aim of our study was to explore the sources of satisfaction at work. Promoting job satisfaction is important for educators and for the provision of optimal quality care in health and social services (Corr et al., 2014). Job satisfaction plays a crucial role in educators’ work achievement, motivation to work, health and quality of life, both at an individual and organizational level (Malinen and Savolainen, 2016; Pikò and Mihàlka, 2017). In addition, as previously mentioned, job satisfaction is crucial in order to support avoidant educators to feel effective and able to cope with their recurrent work issues.

The synthesis (Contini, 2014) of the two studies offers an original contribution to a quite unexplored field and seeks to provide an initial response to the following question: how is it possible to provide training and support to avoidant professionals in order to enhance their work-related self-efficacy in the context of a helping profession? In study 2 we carried out a qualitative examination of educators’ positive perceptions and satisfying work experiences. Using thematic analysis, we identified the core themes of educators’ job satisfaction.

The first theme is named *Social Interactions at Work*. Several studies highlight that the interpersonal cooperative and supporting relationships with co-workers and supervisors are important factors potentially increasing job satisfaction (Belias and Koustelios, 2014; Gkolia et al., 2014). The relationship with users is essential for youth-care workers (Fein, 2014; Frøyland, 2018).

The user/professional relationships provide educators with internal rewards and gives meaning to their work (O’Keeffe et al., 2015; McCallum et al., 2017), they are fulfilling (Berlanda et al., 2017) supporting thereby workers’ high intrinsic job satisfaction (Cameron, 2003; Collins, 2008). Working with clients is often source of a sense of self-actualization (Papadaki and Papadaki, 2006). In child welfare, especially within residential home services, educators operate more closely and engage more frequently with the children. These relationships represent the context in which people-changing activities take place, requiring educators’ professional understanding and social skills (Graham and Fulcher, 2017). When working conditions become increasingly challenging, the support of colleagues and supervisors becomes an aspect that can help overcome crises affecting performance and job satisfaction (Del Valle et al., 2007). Relation with colleagues and supervisors is based on how employees perceive their specific work-related interactions (Lizano and Mor Barak, 2012). The quality of relationships differs from one institution to another because it is largely determined by the management/leadership style. Supportive and caring leaders and the authentic leader-follower relationship (Hinojosa et al., 2014) typically increase employees’ satisfaction and performance (Chen and Scannapieco, 2010). Support from coworkers improves job satisfaction (Papadaki and Papadaki, 2006; McFadden et al., 2015) and, a fortiori, positive messages delivered by supervisors improve worker’s self-esteem and self-efficacy (Gibbs, 2001).

The second theme is *Competence and Care*. Residential child-care workers engage on a daily basis in relationships with the children (Zerach, 2013). They are considered the “front-line” professionals in residential child-care (Seti, 2008) since they have the most direct and recurrent interaction with the children (Fein, 2014). Helping users is an important source of pleasure and satisfaction in social services (Ayele, 2017), in these contexts educators are often key support givers for children (Frøyland, 2018) responding to their psychological, emotional and functional needs (Zerach, 2013). The last theme is *Personal Values.* The positive emotional and caring attention for children seems to be the core element of this theme. Educators point out a sense of gratification and fulfillment rooted in the awareness that they are able to develop closeness and a positive warm connection to children. According to Moses (2000) the majority of educators consider their job as a sort of personal fulfillment, as a way of giving and receiving intangible rewards. Their responses reflect a high degree of ideological and psychological motivations to work in residential child care. According to Colton and Roberts (2007), and Rycraft (1994), a sense of personal “mission” represents a key factor of educators’ satisfaction at work.

Findings of study 2 suggest three areas in which managers could intervene to develop, support and monitor strategies and interventions in line with the professionals’ need to increase job satisfaction. The most prominent one represents issues and themes related to satisfying cooperative co-working experiences and supportive supervision practices. A better work experience is helpful for the educators and for the children they care for.

According to literature, both dispositional and contextually activated attachment avoidance lead to inhibition or interference with any type of communal or cooperative orientation (Mikulincer and Shaver, 2007). Moreover, attachment avoidance seems to reduce responsiveness to others’ needs. In line with these findings our results show that attachment avoidance is a precursor of low self-efficacy. Nevertheless, we also demonstrated that a personal experience of job satisfaction allows avoidant professionals to perceive higher levels of work-related self-efficacy. Supervisors and third sector service-management could implement our results in their adult and continuous-training activities for educators, in order to enhance their supportive and cooperative perspectives, competences and behaviors at work, thereby empowering them toward higher levels of job satisfaction in the most prominent identified area.

We recognize some limitations of our studies. Firstly, the cross-sectional nature of our research. In addition, ours is a sample of convenience, and deliberately focusing on a group of workers under greater than normal stress. Moreover, we rely exclusively on self-reports, and participants may not necessarily be aware of their perceptions and thoughts or may respond in a socially desirable way. Another limitation of study 1 is its correlational nature. Study 2 was carried out with a qualitative methodology and this surely affects the generalizability of our results.

Further studies should aim to analyze applying a quantitative methodology the extent to which avoidant and anxious professionals share/or differ in their evaluation of/the three identified sources of educators’ satisfaction at work. Also, additional studies should focus on different samples of professionals not exposed to extremely high level of stress.

## Author Contributions

All authors listed, have made substantial, direct and intellectual contribution to the work, and approved it for publication.

## Conflict of Interest Statement

The authors declare that the research was conducted in the absence of any commercial or financial relationships that could be construed as a potential conflict of interest.
